# Comparative transcriptomic analysis of retinal response to diverse cellular stresses reveals relative contributions of different cell death processes and signalling networks

**DOI:** 10.1038/s41419-025-08257-w

**Published:** 2025-12-01

**Authors:** Subhradeep Sarkar, Ramaraj Kannan, Trailokyanath Panigrahi, Karthickraja Veeramani, Thirumalesh MB, Shom Shanker Bhattacharya, Arkasubhra Ghosh

**Affiliations:** 1 GROW Research Laboratory, Narayana Netralaya Foundation, Bangalore, Karnataka India; 2 Manipal Academy of Higher Education, Manipal, Karnataka India; 3 Narayana Nethralaya Eye Hospital, Bangalore, Karnataka India; 4 Division of Molecular Genetics, Institute of Ophthalmology, University of London, London, UK

**Keywords:** Cell death, Transcriptomics

## Abstract

Retinal degeneration comprises a diverse group of progressive disorders leading to visual impairment and ultimately blindness. These include inherited retinal dystrophies (IRDs), diabetic retinopathy (DR), age-related macular degeneration (AMD), and glaucoma, affecting millions worldwide. The underlying pathology involves dysfunction and death of photoreceptor cells and the retinal pigment epithelium (RPE), driven by various stress-induced cell death mechanisms. Although multiple pathways have been reported, the relative contribution of each remains incompletely understood, highlighting the need for further investigation. Therefore, we studied how different stress types that induce retinal degeneration alter the global gene expression profile in vivo (C57BL/6 mice), aiming to identify predominant cell death mechanisms as well as key genes and networks. Retinal toxicity was induced using established models of oxidative stress, hypoxia, endoplasmic reticulum (ER) stress, and chronic inflammation. Transcriptomic profiling revealed both unique and convergent gene expression changes associated with each stressor. In total, 170, 328, 146, and 151 genes were significantly altered under oxidative stress, inflammation, ER stress, and hypoxia, respectively (Log2 fold change >2 or <-2; *p* < 0.05). Genes such as Arhgap26, Ccdc9, Ube2e2, and Fndc3b were commonly dysregulated across ER stress, inflammation, and oxidative stress, whereas Nfix, Elp6, Naca, and Plcd3 were selectively altered in oxidative stress, inflammation, ER stress, and hypoxia, respectively. Analysis of cell death-related gene subsets revealed that pyroptosis was commonly activated across different stress types. Additionally, autophagy-mediated cell death, ferroptosis, and extrinsic apoptosis were preferentially associated with oxidative stress, chronic inflammation, and hypoxia, respectively. Both ER and oxidative stress models showed strong activation of autophagy-associated cell death. Together, these findings delineate distinct molecular signatures and predominant cell death mechanisms triggered by specific stressors, providing important insights that could aid in developing targeted therapies to prevent or slow retinal degeneration.

## Introduction

Degeneration of the highly specialized retina layers underpins the primary cause of vision loss and irreversible blindness in millions worldwide. Retinal cell death and remodeling occur across diseases such as age-related macular degeneration (AMD) [1], diabetic retinopathy [2], glaucoma [3], and inherited retinal dystrophies (IRDs) such as retinitis pigmentosa, Stargardt disease, etc [4, 5]. The visual cycle is a finely calibrated process that occurs cells within the photoreceptors and retinal pigment epithelium (RPE), the output being connected to the brain via the bipolar cells and ganglion cells to the optic nerve and to the brain, which are supported by the other retina-specific cell types and the choroid [6]. While diverse stresses have been identified as the drivers of pathologies distinctive within each type of retinal disease, and specific disease pathways are being studied, they usually converge on the common cell death pathways, resulting in loss of PR and/or RPE [7]. Various animal models, particularly mouse models, have been employed to understand the molecular signaling networks underpinning mechanisms driving loss of vision. Cumulatively, such studies indicate that within the retina, there are likely multiple cell death mechanisms operational under any given stress. Hence, we investigated, using a few major, well-known retinal degeneration-inducing stress stimuli, the response of the mouse retina. Such a comparative common approach improves and expands our understanding of the preferential retinal response in the context of cell death mechanisms in vivo.

Earlier reports suggested that apoptosis was the main cell death mechanism for rod, cone, and photoreceptor cell death [8]. The intrinsic apoptotic pathway triggers the cell death by activating initiating caspases 8/9, which further activates the effector caspases (3, 6 and 7), and nuclear damage occurs [9]. The extrinsic apoptotic pathway is activated by TNFα and Fas ligands upon bound to the death receptors on the surface of the damaged cell [9]. Since the lack of disease management upon inhibition of caspase suggested the involvement of non-apoptotic cell death mechanisms in this degenerative process such as regulated necrosis (necroptosis [10], pyroptosis [10, 11], ferroptosis [12], parthanatos [10] and paraptosis), autophagy [13], and cyclic guanosine monophosphate (cGMP) dependent photoreceptor death [14]. These cell deaths in IRDs were triggered by oxidative stress [15], inflammation [16] and endoplasmic reticulum (ER) stress [17] through a unique mechanism of action. For instance, necroptosis is induced by the TNFα pathway and mediated by protein kinase-1 receptor (RIPK1) and RIPK3 complex [18], pyroptosis gets activated by pro-inflammatory caspases [19]; parthanatos gets triggered by the activation of poly-ADP-ribose polymerase (PARP) and Ca^2+^ dependent cystine proteinases (calpains) upon oxidative stress or inflammation [20]; iron overload enhances oxidative stress-induced ferroptosis [12]. There are series of molecular events that regulate each of these mechanisms in a sequential manner.

The study on retinal degeneration heavily relies on the use of mouse models. Compared to genetic models, the severity of the degeneration and the onset of the disease can be controlled in induced models. Further, to study the effect of different stress conditions that trigger cell death, induced mouse models hold the advantage over the inherited genetic mouse models. Extensive research on photoreceptor cell death upon various stress conditions such as oxidative stress, hypoxia, inflammation and ER (endoplasmic reticulum) stress could be useful in studying the underlying mechanisms.

We hypothesize that distinct pathological stressors—oxidative stress, hypoxia, ER stress, and inflammation—contribute to photoreceptor and RPE degeneration through unique but interconnected molecular pathways. Specifically, sodium iodate (NaIO₃)-induced oxidative stress triggers caspase-dependent apoptosis and ferroptosis [21, 22], cobalt chloride (CoCl₂) mimics hypoxic conditions leading to HIF-1α-mediated photoreceptor dysfunction [23], tunicamycin-induced ER stress activates the unfolded protein response (UPR) culminating in CHOP-dependent apoptosis [24], and lipopolysaccharide (LPS)-mediated inflammatory stress amplifies neuroinflammation and microglial activation, resulting in retinal degeneration [25]. By systematically investigating the molecular signatures and regulatory pathways involved in these stress-induced cell death mechanisms [26]. This study aims to elucidate the underlying pathophysiology of retinal degeneration and identify potential therapeutic targets to mitigate photoreceptor loss in IRDs and other retinal diseases.

## Methodology

### Animal husbandry, the induction of retinal degeneration and treatments

#### Ethics approval and consent to participate

C57BL/6 mice were sourced from The Jackson Laboratory. All animal experiments were conducted in accordance with the Association for Research in Vision and Ophthalmology (ARVO) Statement for the Use of Animals in Ophthalmic and Vision Research. The study protocol (Project No. ABD/IAEC/PR/324-24-25) was reviewed and approved by the Institutional Animal Ethics Committee (IAEC) of Anthem Biosciences Pvt. Ltd.

#### C57BL/6 mice animals

C57BL/6 mice were maintained under a 12-h light/dark cycle with unrestricted access to food. All the mice were 5–6 weeks old and weighed between 17 to 20 g. Only male mice were included in the study due to their availability at the time of acquisition, However, existing research indicates that sex differences do not significantly affect RPE or photoreceptor damage in this context. For all experimental groups, *n* = 3 mice per group were used, and each experiment was independently repeated to ensure reproducibility.

#### Intravitreal injection into mice

All the animals were anesthetized using ketamine-xylazine via intraperitoneal (IP) injection prior to the treatment. The standard IP dosage for anesthesia was ketamine (80–100 mg/kg) and xylazine (5–10 mg/kg) to ensure adequate sedation before the procedure. The procedure began with a nasal corneal puncture approximately 0.5 mm medial to the dilated pupillary margin, using a 30-gauge hypodermic needle to access the anterior chamber. A 33-gauge blunt needle (Hamilton Company, NV) was then carefully inserted through the corneal puncture, ensuring minimal trauma to the iris and lens. The needle was advanced laterally from the iris and medially toward the lens, displacing the lens medially as it progressed through the vitreous cavity to the retinal surface. Once positioned at the intended injection site, 1.2 μl of cobalt chloride (12 nmol/eye, Sigma, Cat. no: C8661-25G), 1 μl of LPS (100 µg/eye, Sigma Aldrich, Cat. No: L6143-1MG), and 1 μl of tunicamycin (10 nmol/eye, Sigma Aldrich, Cat. No: T7765-1MG) solution were slowly injected via intravitreal injection (IVI), with the needle held in place for approximately 30 s to ensure proper diffusion.

#### Intravenous injection to mice

Sodium iodate (NaIO₃, 35 mg/kg; Sigma-Aldrich, CAS No: 7681-55-2) was freshly prepared by diluting in sterile 1X PBS and filter sterilized immediately before use. The 100 µL solution was then administered via tail vein injection to the animals under appropriate handling conditions.

### Tissue harvesting

5% isoflurane was maintained in the Induction chamber for one minute until the animals underwent respiratory arrest to euthanize them. Following enucleation, OCT blocks of the eyeballs were prepared for histological analysis. Additionally, the retina of the contralateral eyes was dissected out for RNA extraction.

### Optical coherence tomography and fundus imaging

Optical coherence tomography (OCT) measurements were performed to assess progressive retinal structural changes and total retinal thickness over time following the induction of various pathological stresses. A commercially available Ocuscience system, modified for animal use, was utilized for imaging. Fundus imaging was conducted to identify retinal abnormalities, including vascular attenuation and increased retinal pigment epithelium (RPE) pigmentation. Prior to imaging, mouse pupils were dilated using 0.8% Tropicamide with Phenylephrine Hydrochloride ophthalmic solution, and light intensity was carefully adjusted to prevent overexposure.

### Tissue TUNEL staining

The Terminal deoxynucleotidyl transferase dUTP nick end labeling (TUNEL) assay was performed using the TUNEL Assay Kit (Elabscience; Cat. No.: E-CK-A320) following the manufacturer’s protocol. Briefly, frozen sections of the whole mouse eye globes, which was subjected to different pathological stresses were thawed at room temperature (RT) and fixed with 4% paraformaldehyde (PFA). The samples were then incubated in terminal deoxynucleotidyl transferase (TdT) equilibration working buffer for 30 min at RT, followed by treatment with TdT enzyme working solution for 30 min at 37 °C in a humidified chamber. The nuclei were counterstained with DAPI and the slides were sealed using a mounting medium for further analysis. The imaging was acquired using an upright fluorescent microscope (Olympus CKX53).

### Histopathology and light microscopy

The Optimal Cutting Temperature embedded tissue samples were sectioned at 10 µm thickness and stained with hematoxylin and eosin (H&E) for histomorphological analysis. Frozen slides were equilibrated to room temperature and fixed in ice-cold methanol for 10 min. This was followed by sequential processing in 95% alcohol for 5 min and rinsing in distilled water. The tissue sections were immersed in hematoxylin solution for 4–5 min, followed by rinsing in water and differentiation using 1% acid alcohol. The slides were then washed under running tap water for 4 min. For eosin staining the tissue sections were immersed in eosin for 30 s to 1 min. followed by gentle washing in running tap water. The samples were then dehydrated in 95% alcohol for 2 min, cleared in Xylene I and Xylene II (2 min. each), and mounted using a mounting medium. The brightfield images were captured using an Olympus CKX53 microscope for further analysis.

### RNA extraction, quantitative real-time PCR (qRT-PCR) analysis

Total cellular RNA was extracted from contralateral eyes (*n* = 3 per experimental group) using TRIzol reagent (Nucleospin; MN-740955.50) following the manufacturer’s protocol and RNA concentration was determined using a spectrophotometer. Quantitative real-time PCR (qPCR) was performed using cDNA converted wit BioRad iSCRIPT cDNA synthesis kit (BioRad, USA) following the manufacturer’s instructions. A total of 1000 ng of the synthesized cDNA was used for gene expression analysis. The mRNA expression levels of target genes were normalized to 18S.

### RNA-sequencing

The total RNA was isolated from the retina using RNeasy Mini Kit (Cat No: 74104). The library preparation was performed for 1 ug of total RNA using NEBNext RNA Ultra protocol. The rRNA from the cytoplasm and mitochondrial origin were removed using biotinylated beads. Further the RNA was fragmented using divalent cations at higher temperatures. Further, cDNA was synthesized using reverse transcriptase. The generated cDNA was subjected to series of enzymatic reactions for end repair, A-tailing and ligation with adapters. The prepared libraries were sequenced as paired-end on Illumina HiSeqX for 60 M reads with 150 bp reads per sample.

### NGS data analysis

The generated sequence reads were subjected to quality check using FASTQC. The adapter reads were trimmed using Trimmomatic tool. Furthermore, the sequence reads were aligned with GrCM39 using the STAR aligner. The aligned reads were annotated using STRINGTIE2. Further, the CPM based normalization was performed using EdgeR. The normalized reads were used for further analysis.

### Data analysis

R programming was used for data visualization and data analysis. Heatmap was generated using Complex heatmap library in R. Student’s T-test was performed using Scipy package in Python 3.7.

### Data retrieval for meta-analysis

The transcriptomics studies on various retinal degenerative diseases such as AMD, FKBP/CASP8-mediated apoptosis model in RPE cells, DOTL1 gene knockout in retinal microvascular endothelial cells and RP-derived iRPE cells were retrieved from GEO (Gene Expression Omnibus) database under the accession GSE115828, GSE297557, GSE298743, and GSE271751 respectively. Also, the non-primate models to understand the molecular mechanisms underlying retinal degeneration such as zebra fish model for Choroideremia, glaucoma model in C57BL/6 mice, Baml1 knockout in microglia and retinal cells of C57BL/6 mice, DR model in db/db mice and hyperoxia induced model in C57BL/6 mice were shortlisted from the accession number GSE254948, GSE297955, GSE297955, GSE282215 and GSE261490, respectively.

## Results

### In vivo evaluation of retinal morphology, functional integrity of the retinal vasculature and fundus imaging in pathological stress-induced C57BL/6 mice

**Fig. 1:**
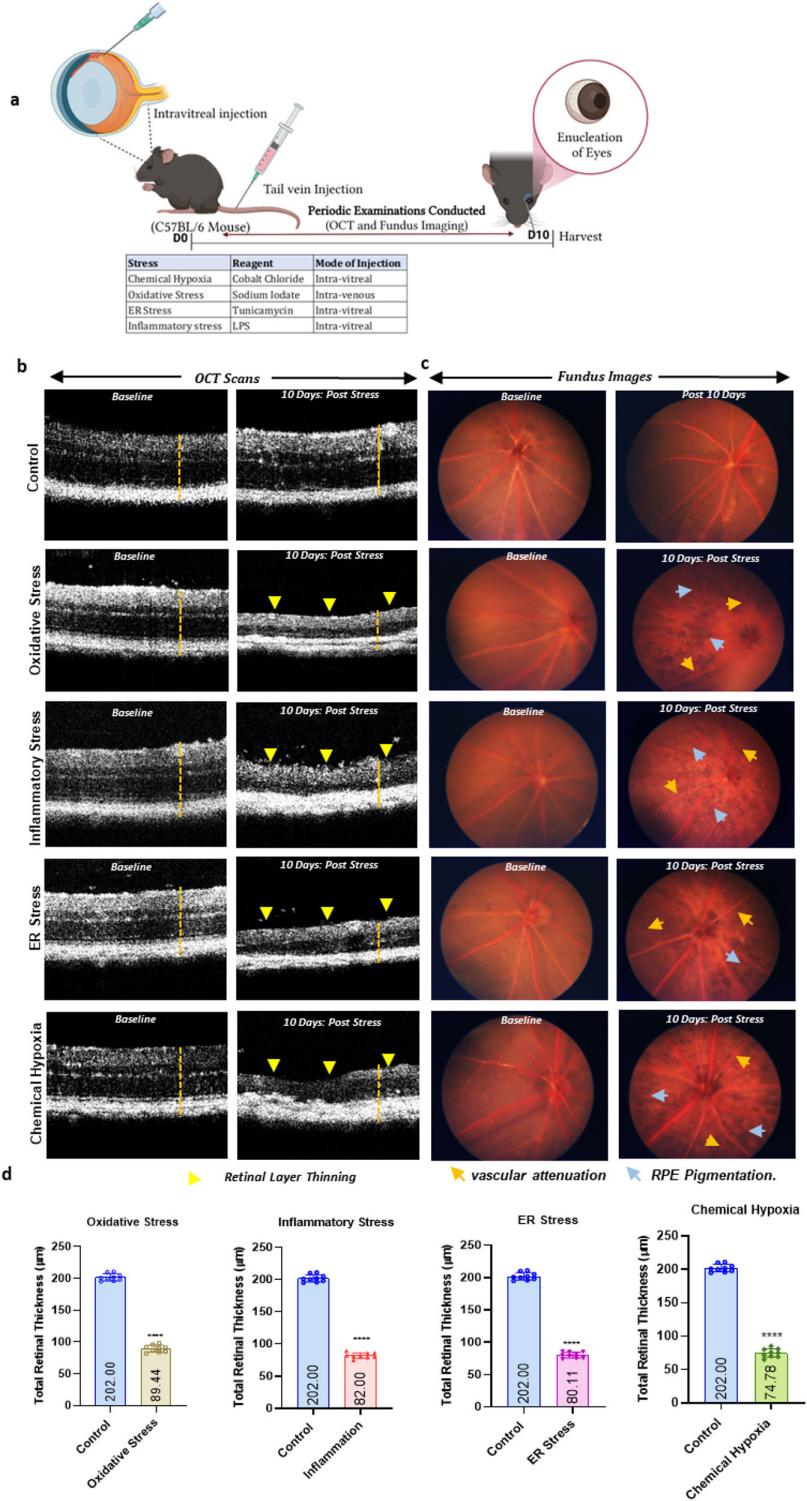
Reduced photoreceptor survival in retinas subjected to pathological stress compared to PBS-injected control eyes. **a** Schematic representation of experimental induction of various pathological stresses in the retina using a murine model (*n* = 3). **b**, **d** Representative optical coherence tomography (OCT) images showing progressive retinal structural changes & total retinal thickness over time following induction of various pathological stresses. (yellow) Arrowheads mark regions of retinal disruption. **c** Representative fundus images showing retinal vascular and structural features under different pathological stress conditions. Arrowheads mark regions showing retinal abnormalities, including (mustard yellow) vascular attenuation and (blue) signs of increased RPE Pigmentation. Data and error bars indicate mean ± SD. ns, not significant. *p < 0.05, **p < 0.01, ***p < 0.001, and ****p < 0.0001.

To assess retinal morphology alterations, C57BL/6 mice were subjected to pathological stresses—oxidative stress, ER stress, inflammatory stress, and chemical hypoxia—following the workflow outlined in Fig. 1a. At baseline (Day 1), all mice displayed intact retinal layers. Post-stress induction, mice exhibited significant degeneration and disruption of the photoreceptor layer and retinal pigment epithelium (RPE), with a marked reduction in total retinal thickness compared to controls (chemical hypoxia: 79.0 ± 3.57; inflammatory stress: 81.0 ± 3.611; oxidative stress: 90.0 ± 3.26; ER stress: 80.0 ± 3.72; control: 201.8 ± 3.72; p ≤ 0.0001 for all; Fig. 1b, d). Degeneration progressed over time and was irreversible within the 10-day post-injection period, indicating sustained retinal damage. Control eyes showed no morphological alterations (Fig. 1b).

Fundus imaging further revealed stress-induced vascular abnormalities, including vascular attenuation and increased RPE pigmentation (Fig. 1c), absent in PBS-treated controls. Early vascular attenuation, characterized by reduced vessel density and caliber, suggests ischemia or impaired perfusion contributing to retinal degeneration. Enhanced RPE pigmentation likely reflects stress responses such as oxidative damage or inflammation. Overall, pathological stressors disrupted retinal homeostasis, leading to progressive structural and vascular deterioration.

### Histological assessment of retinal degeneration

**Fig. 2:**
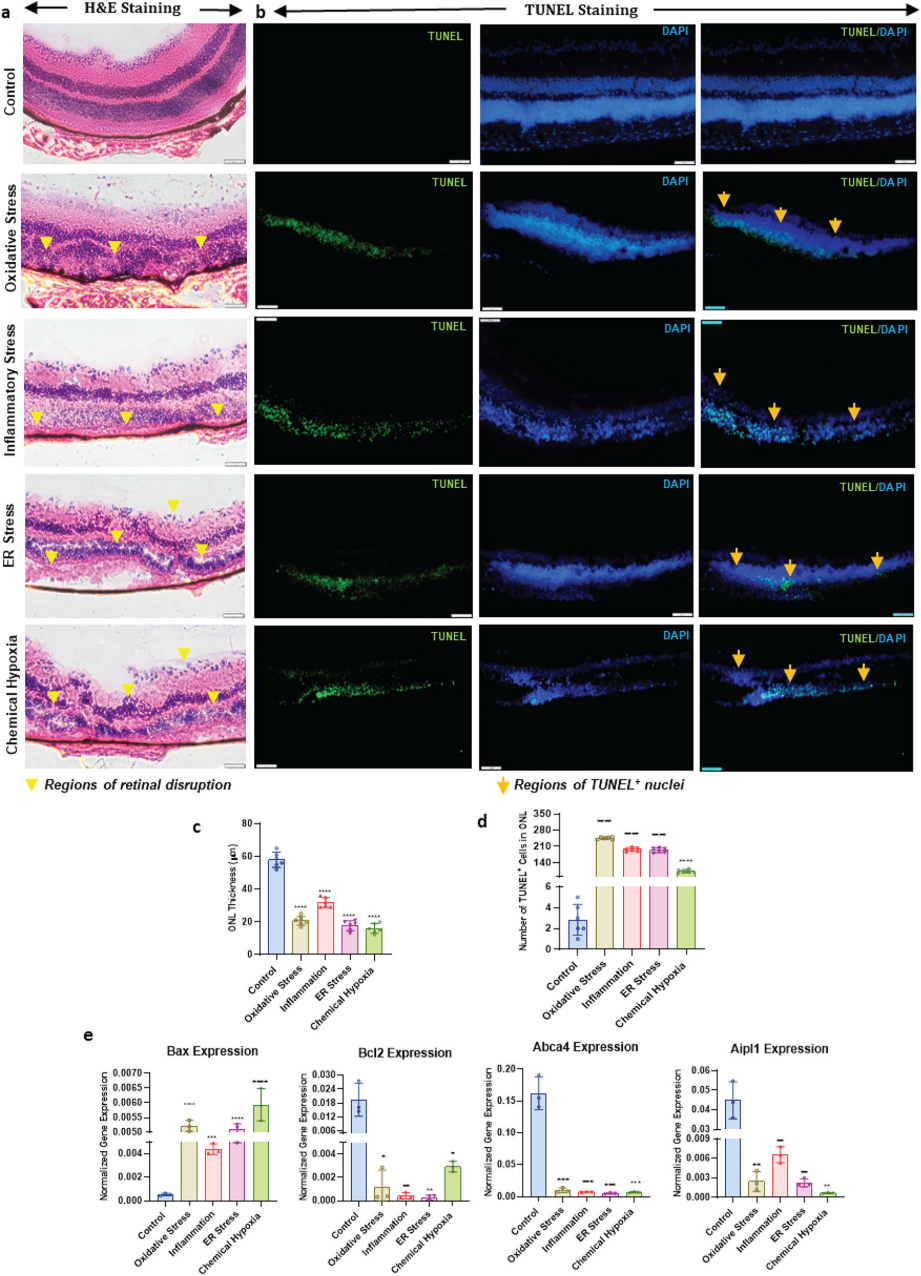
Analysis of retinal degeneration and photoreceptor cell death in pathologically stressed induced retinas versus PBS-injected control eyes. **a** Representative hematoxylin and eosin (H&E) stained retinal cross-sections illustrating progressive structural alterations and changes in total retinal thickness over time in response to various pathological stressors. **b** Representative immunofluorescent images of TUNEL-stained retina during the progression of photoreceptor cell degeneration. Arrowheads mark regions of TUNEL^+^ nuclei. **c** Mean of four fields was used for the ONL thickness quantification (*n* = 3). **d** Mean of four fields was used for the quantitation of the number of TUNEL+ nuclei, represented as the density of TUNEL+ nuclei per 20× field of view e) Quantification of Apoptotic, survival and photoreceptor-specific markers. Data and error bars indicate mean ± SD. ns, not significant. *p < 0.05, **p < 0.01, ***p < 0.001, and ****p < 0.0001.

Photoreceptor degeneration was evaluated by H&E staining of retinas exposed to pathological stresses. Compared to PBS controls with intact layers (Fig. 2a, c), stressed retinas showed layer thinning and structural disruption. Chemical hypoxia and ER stress reduced INL, ONL, and GCL thickness, with ER stress also causing neural retinal detachment. Inflammatory stress led to widespread thinning and photoreceptor loss, while oxidative stress caused severe ONL/INL thinning, complete photoreceptor degeneration, and RPE disruption. A consistent dosage and harvest time were used to standardize comparisons across groups, although distinct pathways and cellular responses emerged depending on the stressor, as confirmed by transcriptomic analysis.

### Assessment of apoptosis in retinal degeneration

TUNEL staining revealed stress-induced nuclear DNA fragmentation in the ONL and other retinal layers (Fig. 2b). TUNEL+ cells significantly increased across all stress conditions, indicating elevated photoreceptor apoptosis (Fig. 2b, d). Oxidative stress induced the highest TUNEL+ count (242.8 ± 2.207, *p* ≤ 0.0001), followed by ER stress (190.8 ± 4.009), inflammatory stress (195.3 ± 3.585), and chemical hypoxia (100.3 ± 3.075), compared to minimal staining in controls (2.75 ± 4.58, *p* ≤ 0.0001).

To corroborate these findings, we analyzed the expression of Bax, Bcl2, and photoreceptor-specific genes ABCA4 and AIPL1 (Fig. 2e). Expression of Bcl2, Abca4, and AIPL1 was significantly downregulated under inflammatory (Bcl2: 0.00042 ± 0.0041, *p* ≤ 0.009; Abca4: 0.00749 ± 0.0145, *p* ≤ 0.0005; Aipl1: 0.0065 ± 0.0055, *p* ≤ 0.0023), oxidative (Bcl2: 0.0012 ± 0.0042, *p* ≤ 0.0012; Abca4: 0.00984 ± 0.0150, *p* ≤ 0.0005; Aipl1: 0.0024 ± 0.0055, *p* ≤ 0.0016), ER stress (Bcl2: 0.00028 ± 0.0041, *p* ≤ 0.0096; Abca4: 0.00489 ± 0.0149, *p* ≤ 0.0005; Aipl1: 0.00228 ± 0.0055, *p* ≤ 0.0015), and chemical hypoxia (Bcl2: 0.00029 ± 0.0041, *p* ≤ 0.0016; Abca4: 0.00689 ± 0.0149, *p* ≤ 0.0005; Aipl1: 0.00550 ± 0.00068, *p* ≤ 0.0013). Conversely, Bax expression was significantly elevated across all stress conditions, with chemical hypoxia inducing the highest levels, indicative of robust apoptotic activity. In contrast, control samples showed higher Bcl2, Abca4, and Aipl1 expression, suggesting preserved photoreceptor integrity.

### Transcriptomics analysis of stress-induced retinal models in mice

**Fig. 3:**
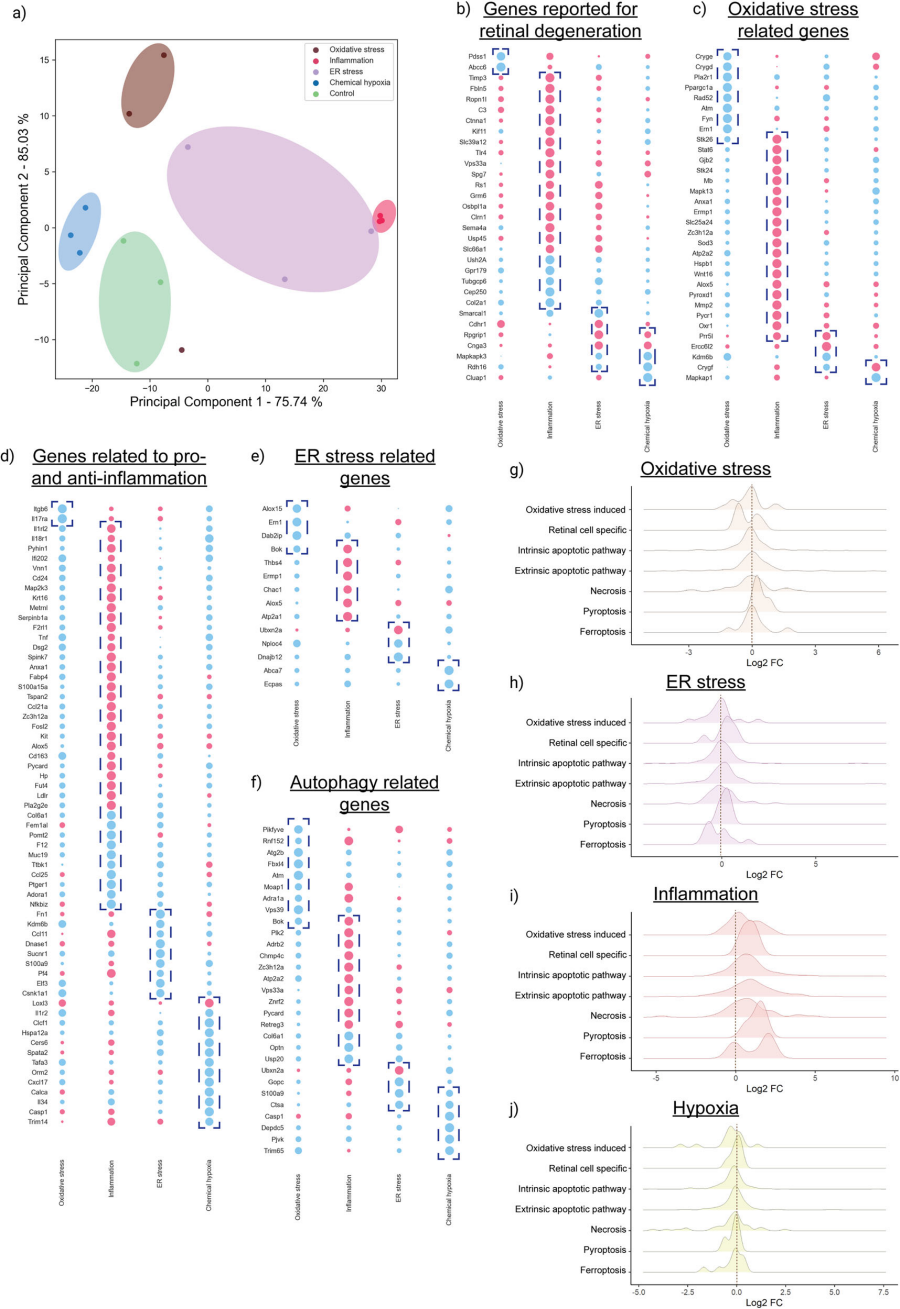
Transcriptomics analysis of stress induced retina. **a** Principal component analysis (PCA) shows the distinct expression profile for each stress conditions, whereas the control and inflammatory groups formed a distinct clusters. **b**–**f** Distinct gene expression profile for the genes involved in IRD disease, autophagy, inflammation, ER stress and oxidative stress respectively. **g**–**j** Density plot shows that the expression pattern for each type of cell death mechanisms such as apoptosis (intrinsic and extrinsic), pyroptosis, ferroptosis, oxidative stress induced apoptosis, retinal cell specific apoptosis and necrosis for the different stress conditions such as oxidative stress, inflammation, hypoxia and ER stress induced conditions respectively.

Unbiased transcriptomics profiling was performed on the retina of stress-induced C57BL/6 mice. Principal Component Analysis (PCA) was carried out after filtering out low-abundant genes. Three distinct gene expression clusters were observed for chemical hypoxia, inflammation, and PBS control models (Fig. 3a). A large variance was seen in oxidative stress models. Over 900 genes associated with retinal degenerative diseases were evaluated across different stresses. Oxidative stress caused downregulation of Pdss1 and Abcc6, known to be involved in retinitis pigmentosa and retinopathy. Inflammatory stress upregulated cell adhesion (Spg7, Ctnna1, Fbln5) and visual perception genes (Clrn1, Grm6, Rs1), while genes related to visual maintenance (Gpr179, Col2a1, Ush2a) and mitotic division (Cep250, Tubgcp6) were downregulated. Retinal development genes like Rpgrip1 and Cnga3 were also affected. Under hypoxic stress, retinoic acid metabolism genes Mapk3, Rdh16, and Clua1 showed reduced expression (Fig. 3b).

Gene Ontology (GO) annotations related to oxidative stress (*n* = 187), inflammation (*n* = 596), ER stress (*n* = 226), and autophagy (*n* = 412) were analyzed. Fold change patterns revealed that oxidative stress downregulated genes such as Ppgarc1a, Rad52, Atm, Pla2r1, Fyn, Ern1, and Stk26, whereas inflammatory stress upregulated Stat6, Gjb2, Stk24, Mapk13, Anxa1, and Ermp1, and ER stress models upregulated Ercc6l2 and Prr5l (Fig. 3c). Crystallin proteins Crygd, Cryge, and Crygf, known for responding to reactive oxygen species, were downregulated under oxidative stress but upregulated under hypoxia. Hif1α expression remained unaltered. Inflammatory response genes were highly expressed during inflammation but suppressed under oxidative, ER, and hypoxic stress (Fig. 3d). ER stress-induced apoptotic pathways were activated during inflammation, while ERAD pathway genes (Ubxn2a, Nploc4, Dnajb12, Ecpas) were downregulated under other stresses (Fig. 3e). Oxidative stress, ER stress, and hypoxia models showed reduced expression of autophagy inhibitory genes, while inflammatory stress promoted autophagosome maturation and assembly (Fig. 3f).

To understand the mechanisms of photoreceptor loss, key genes associated with ferroptosis (*n* = 11), pyroptosis (*n* = 4), necrosis (*n* = 41), intrinsic and extrinsic apoptosis (*n* = 162, 203), oxidative stress-induced death (*n* = 16), autophagy-mediated death (*n* = 13), and retinal-specific markers (*n* = 7) were analyzed. Density plot analysis based on log2 fold changes indicates that oxidative stress upregulated pyroptotic genes and ferroptosis (Fig. 3g). ER stress induced pyroptotic cell death and ferroptosis (Fig. 3h). Inflammatory stress upregulated almost all types of cell death pathways, with a prominent increase in pyroptosis and ferroptosis-associated genes (Fig. 3i). Hypoxic stress did not show a clear differential pattern between the pathways (Fig. 3j). Thus, we find that in each of the stresses, most of the cell death pathways are operational at the whole retina level, yet the relative contributions in each are different. Overall, pyroptosis emerged as a favored, commonly induced, cell death pathway across all stresses, with hypoxic stress inducing several pathways almost to a similar extent.

### High abundant gene expression

**Fig. 4:**
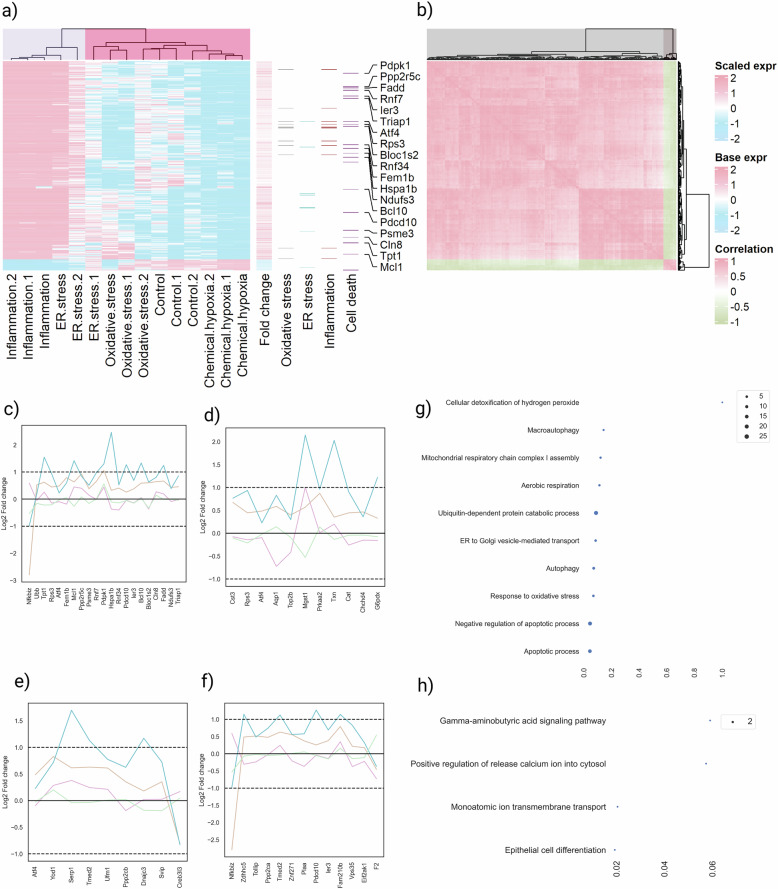
Most abundant gene expression. **a** The heatmap shows the gene expression for top 515 abundant genes shortlisted based on normalized FPKM values. The second panel of the heatmap shows the fold change of the stress-induced models to the control mice. The third panel shows the involvement of genes which involve in the oxidative stress, ER stress, inflammation and cell death mechanism. **b** The Spearman correlation matrix of the abundant genes. The hierarchical clustering analysis found two distinct clusters. **c**–**f** The Log2 fold change values of the genes involved in the cell death, ER stress, oxidative stress and inflammation respectively. **g** The bubble plot shows the major biological process for the genes in the cluster 1. The size of the dot represents the number of genes identified. **h** The bubble plot shows the major biological process for the genes in cluster 2.

After stringent filtering, 25,528 genes were retained. Genes with low detection frequency were removed, and Spearman’s rank correlation was performed. Genes with absolute correlation >0.7 across more than 1000 genes were shortlisted, resulting in 515 high-abundant genes. Hierarchical clustering showed inflammation models grouped, followed by ER stress models, while hypoxia and control samples clustered separately (Fig. 4a). The second heatmap panel indicated cumulative fold change relative to controls, and the third panel maps high-abundance genes to stress responses and cell death pathways.

Further clustering divided these 515 genes into two distinct groups (Fig. 4b). Cluster 1 (488 genes) was enriched for hydrogen peroxide response, anaerobic respiration, autophagy, proteasome-mediated catabolism, oxidative stress response, and apoptosis regulation (Fig. 4g). Cluster 2 (27 genes) was involved in epithelial differentiation, amino-butyric acid signaling, monoatomic ion transport, and calcium signaling (Fig. 4h). Strong inverse correlation was observed between the two clusters.

Fold change analysis showed that inflammation-related genes were upregulated upon LPS treatment (Fig. 4c), ER stress-related genes were downregulated under hypoxia (Fig. 4d), and oxidative stress models displayed increased inflammatory gene expression (Fig. 4e–f).

### Unbiased differential gene expression analysis

**Fig. 5:**
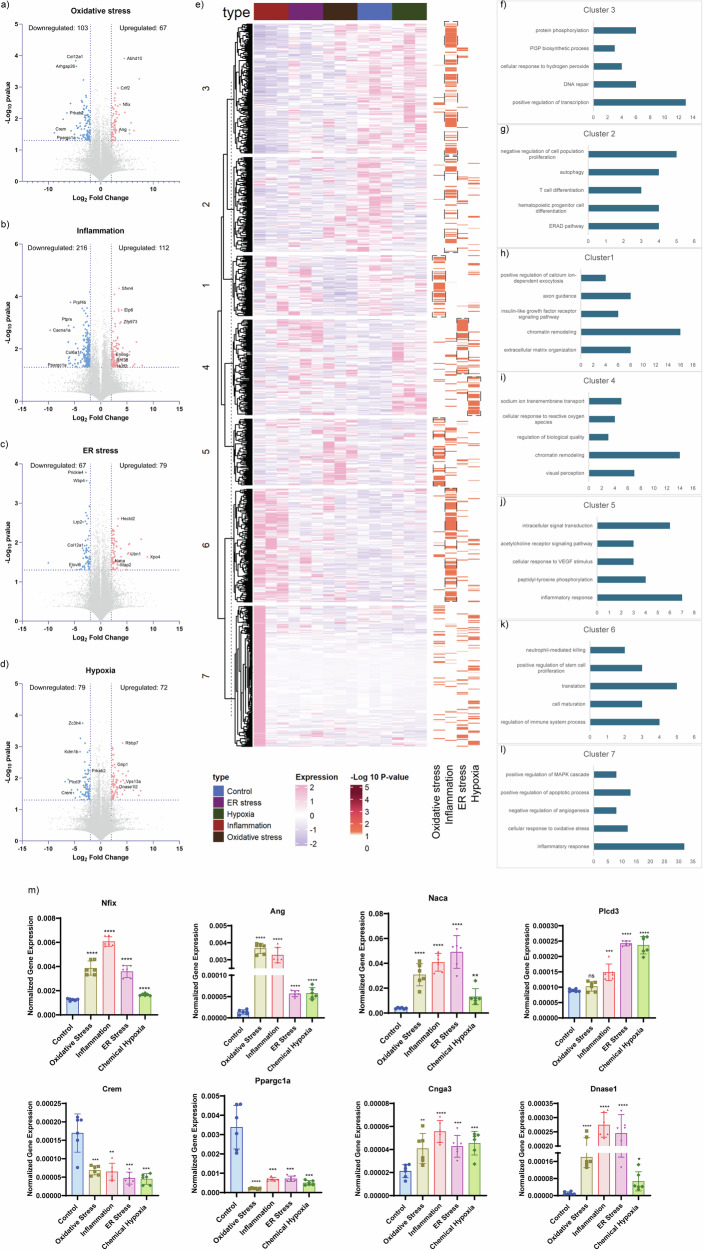
Differential gene expression analysis. **a**–**d** The volcano plot shows the differential gene expression observed under each stress conditions with the *p* < 0.05 and Log_2_ fold change >2 and <-2. **e** Heatmap shows the cluster based on the euclidean distance formed for the DGE. **f**–**l** Major biological process observed for each cluster. **m** The gene expression analysis performed to validate the genes identified in the current study.

Log2 fold changes were calculated for each stress model (*n* = 3) versus PBS control (*n* = 3), with significance assessed by Student’s T-test (*p* < 0.05). Genes with log2 fold change >2 or <-2 were considered significantly up- or downregulated (Supp Table 1). Oxidative stress resulted in 67 upregulated and 103 downregulated genes (Fig. 5a). Enrichment analysis showed that upregulated genes were involved in negative regulation of apoptosis, nitric oxide biosynthesis, and hypoxia response, while downregulated genes related to oxidative stress response, calcium ion transport, and transcriptional regulation (Supp Fig. 1a).

Inflammatory stress upregulated 112 and downregulated 216 genes (Fig. 5b), impacting protein ubiquitination, neutrophil-mediated killing, and transcription regulation, while suppressing ECM organization and chromatin remodeling (Supp Fig. 1b). ER stress led to 67 upregulated and 103 downregulated genes (Fig. 5c), affecting wound healing, ROS response, and microtubule dynamics (Supp Fig. 1c). Hypoxia induced upregulation of genes related to NK cell differentiation and ROS metabolism while downregulating ERAD and apoptotic pathways (Fig. 5d, Supp Fig. 1d).

A combined list of 861 genes (687 significant + 174 relevant but non-significant) was compiled and clustered into seven groups based on Euclidean distance (Fig. 5e). Cluster 1 (166 genes) showed enrichment for calcium exocytosis, IGF signaling, chromatin remodeling, axon guidance, and ECM regulation, with higher significance in oxidative stress models (Fig. 5h). Clusters 2, 3, and 6 were predominantly linked to inflammatory stress, involving autophagy, T-cell differentiation, hydrogen peroxide response, PI3P biosynthesis, and neutrophil activity (Fig. 5g, h, k). Cluster 4 was enriched in ROS response, visual perception, and sodium ion transport, mainly in ER and hypoxia models (Fig. 5i). Cluster 7 contained genes commonly dysregulated across stress models, involving angiogenesis, MAPK signaling, and oxidative stress response (Fig. 5l). Detailed gene-specific expression is summarized (Table 1 and Fig. 5m).

**Table 1:** Differentially expressed genes: The list of commonly DEGs and unique DEGs for each stress conditions with the *p* < 0.05 and Log2 Fold change value > 2 or <-2 were shortlisted.

	Log 2 fold change				-Log 10 *p*-value			
	Oxidative stress	Inflammation	ER stress	Chemical hypoxia	Oxidative stress - *p*	Inflammation - *p*	ER stress - p	Chemical hypoxia - p
Arhgap26	−4.67	−2.28	−2.96		3.66	1.77	2.54	1.85
Ccdc9	−2.25	−6.21	−2.36		1.36	2.26	1.48	
Ube2e2	−4.27	−2.52	−3.48		1.72	1.59	1.85	
Fndc3b	2.15	2.12	2.36		1.44	1.82	1.36	
Ppargc1a	−4.43	−2.42			1.39	1.38		
Crem	−3.69			−5.22	1.66			1.53
Prkab2	−6.44			−2.25	2.18			2.23
Nfix	3.67	3.21	2.45		2.45			
Ang	2.38				1.65			
Naca			2.13				1.53	
Plcd3				−3.02	1.95			1.88
Cacna1a	−2.87	−9.66			1.78	2.71		
Kdelr1	4.58	4.88	3.17		2.2			1.34
Ttc21a	7.35	6.38	7.7	7.59	3.26		2.08	1.6
Tusc2		2.51	2.02	2.06		2.09	1.44	1.58
Gtf3c1		2.12	2.11	2.05		2.19	2.28	
Lmo2	2.22	2.83			1.31	1.92		
Cep63	−2.09	−3.53			1.87	3.55		
Itga3	−3.66	−2.47			2.16	2.6		
Wdr95	−2.03	−2.31			2.21	2.03		
Lrp2	−7.18		−3.4		1.98	1.97	2.52	
Dnajc7	2.9	2.25	2.61		2.22		2.05	
Col12a1	−4.8	−2.19	−2.7		3.84		1.93	
Dclk1	2.27	2.88	2.29			2.31	2.33	
Adamtsl2	3.13	2.56	2.92	2.86		1.5	2.03	
Dst		−2.29				1.35		
Scarf1		−2.25		−2.34	1.59	1.35	1.59	1.43

## Discussion

Despite extensive research across diverse disease models, the mechanisms underlying retinal cell death remain incompletely understood. Apoptosis, necroptosis, and ferroptosis have been implicated in retinal disorders such as AMD, RP, and glaucoma. the precise contributions of these pathways to specific upstream stresses or genetic alterations remain unclear. Moreover, the potential cross-talk among death pathways in the retina is not well defined. Given the retina’s limited regenerative capacity, advancing our understanding of cell death signaling is crucial for developing strategies to prevent photoreceptor and retinal pigment epithelium (RPE) loss and to improve therapeutic approaches. Comparative analyses across stress models can reveal both common and divergent mechanisms, offering insights into novel molecular players.

Photoreceptor degeneration represents the final common outcome in retinal degenerative diseases despite wide phenotypic heterogeneity. Several models, including light-induced injury in BALB/c and C57BL/6 mice [27, 28] and monogenic/knockout models such as Pde6b^rd1^, Prph2^rd2^, rd3-rd10 are a few common retinal degeneration models that are widely used for various experimental studies [29]. Also, Prpf31, Tlcd3b, and Ndufs4 [30–33] are a few other monogenic models used to dissect disease progression. Although DRAM2 mutations cause cone-rod dystrophy [34], Dram2 knockout mice show no overt degeneration or increased apoptosis [35]. Unlike human Stargardt disease, the Abca4 knockout mouse model does not degenerate structurally. Hence, blue light or other inducers are necessary to induce toxicity in mouse models [36]. Thus, while monogenic models can precisely mimic disease phenotypes, they often fail to encompass the multifactorial nature of human disease and impose irreversible retinal damage. In contrast, chemically induced models are cost-effective, reproducible, and versatile, capturing a broader range of pathogenic stress responses.

Among the stressors tested, oxidative stress induced by sodium iodate led to the most profound degeneration, characterized by progressive photoreceptor and RPE disruption, retinal thinning, vascular attenuation, and prominent RPE clustering [37–39]. These findings align with previous reports implicating oxidative stress as a major driver in AMD pathogenesis [40, 41], primarily via ROS-mediated mitochondrial dysfunction, lipid peroxidation, ferroptosis [42], and apoptosis [43]. Sodium iodate-treated retinas exhibited the highest TUNEL+ counts, elevated Bax, and decreased Bcl2, Abca4, and Aipl1 expression. Similarly, ER stress induced by tunicamycin caused significant INL, ONL, and GCL thinning, moderate vascular changes, and irregular RPE pigmentation. ER stress, via unfolded protein response (UPR) overload and CHOP activation [24], is implicated in photoreceptor degeneration in RP [44] and diabetic retinopathy [45–48]. Inflammatory stress via LPS injection led to thinning of retinal layers, photoreceptor loss, vascular attenuation, and patchy RPE hyperpigmentation. This stress activates TLR4 signaling and downstream cytokine production promoting neuronal apoptosis [49, 50]. Our findings align with prior studies linking chronic inflammation to retinal degenerative diseases such as RP [51], diabetic retinopathy [52] and AMD. Chemical hypoxia induced by cobalt chloride exposure caused ONL, INL, and GCL degeneration with early vascular attenuation but minimal RPE disruption. Hypoxia stabilizes HIF-1α, leading to metabolic imbalance and apoptosis [53, 54]. Although TUNEL+ counts were lower compared to oxidative or ER stress, Bax expression was markedly elevated, suggesting early cell death activation through apoptosis or pyroptosis or other mode of cell death mechanisms. Hypoxia-driven ischemia remains a key factor in retinal diseases such as diabetic retinopathy, vein occlusion, and glaucoma [55], likely exacerbates retinal degeneration. Although the process of retinal degeneration varies over time and in severity, for comparative analysis, we selected a dosage and harvest time point at which retinal thickness was consistent across all treatment groups. Nonetheless, the specific pathways, cell types, and underlying mechanisms can differ depending on the type of stress, as revealed by the transcriptomic analysis.

**Fig. 6:**
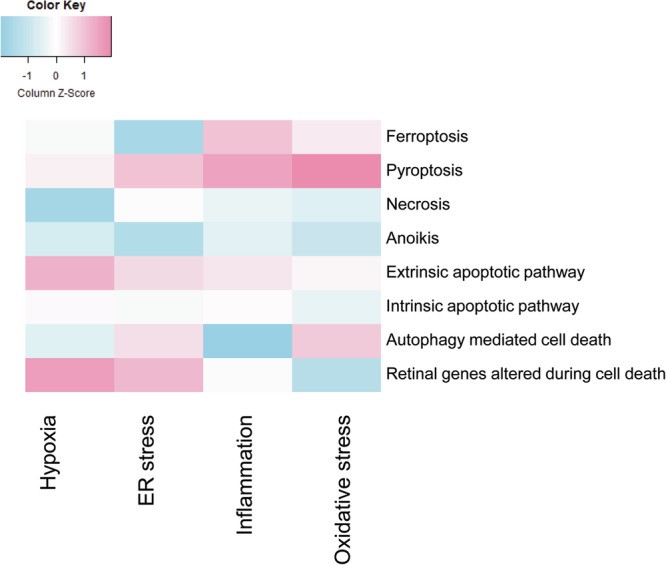
The gene expression pattern for each cell death mechanism under different stress conditions.

Notably, pyroptosis emerged as the predominant cell death pathway with elevated expression in both AMD (GSE115828) and RD12-associated RP (retinal dehydrogenase 12 mutation - GSE271751) (Supp Fig. 2a) which agreed with our data. In contrast, the FKBP/CASP8-mediated apoptosis model in RPE cells demonstrated an inverse expression pattern (GSE297557) with genes associated with retinal specificity, as well as those associated with cell death and necrosis, exhibiting increased expression levels. Furthermore, DOTL1 knockdown in retinal microvascular endothelial cells was associated with activation of the extrinsic apoptotic pathway (GSE298743) (Supp Fig. 2a). Non-primate models used to investigate diverse retinal pathologies were also included in the meta-analysis (Supp Fig. 2b). In an X-linked hereditary retinal degeneration zebrafish model of Choroideremia, elevated expression of pyroptosis-associated genes were found (GSE254948). Similarly, in a C57BL/6 glaucoma mouse model with induced intraocular pressure (IOP), both with and without connexin43 knockdown, pyroptosis-related gene expression was upregulated (GSE297955). Retinal cells depleted of microglial cells exhibited increased pyroptosis gene expression. Hypoxia-induced mouse models also showed elevated pyroptosis gene expression (GSE261490). All these models illustrate agreement with our dataset substantiating our finding that whole retina may utilize pyroptosis as a major cell death mechanism (Fig. 6). However, isolated microglia displayed enrichment of anoikis-associated cell death pathways (GSE282175). In contrast, the db/db mouse model exhibited greater activation of anoikis and intrinsic apoptotic pathway (GSE282215) (Supp Fig. 2b).

The differential gene expression analysis revealed that the 170, 328, 146, and 151 genes were dysregulated upon sodium iodate, LPS, tunicamycin, and cobalt chloride treatments, respectively. A dysregulated list of 867 genes from this study was compared with the transcriptomics analysis of Rd1 mouse model [56], where Jiang et al 2022 attempted to understand the series of molecular changes over 10 days of neonatal. Interestingly, we found 24 gene overlaps with the Pde6b knockout mouse model in the later stage of the knockout. Neonatal P8 and P10 were showing the signs of retinal degeneration. So, we compared neonatal day 8/10 (P8/10) gene expression with P2 and identified that 16 genes (Itga3, Mdm1, Mrps18b, Gnal, Pycr1, Lrp2, Errfl1, Rad52, Ears2, Klf4, Gucy2f, Togaram, Ankrd37, Fam19a3, Ptprz1 and Arfgef2) were following same expression pattern as observed in the current study. This shows that these gene expression changes were observed due to the retinal degeneration and not because of the acute response of the photoreceptor cells under stress conditions.

Arhgap26, Ccdc9, Ube2e2, and Fndc3b were the few genes that were found to be dysregulated under ER stress, inflammation, and oxidative stress. These genes were primarily involved in various regulatory activities in cell signaling. Cacna1a is a neurotransmitter protein that plays a crucial role in voltage-gated calcium ion channels, which are found to be altered under oxidative stress, hypoxia and inflammatory stress. Under ER stress, oxidative stress and hypoxia, Kdelr1 is showing dysregulation. Many gene overlaps were observed between the inflammatory stress and oxidative stress induced mouse models.

NFIX is a transcription factor that comes under the nuclear factor I (NFI) family, which controls cell proliferation, maturation, and migration. NFI family transcription factors are known to be pro-oxidants, hence the elevated expression could lead to oxidative stress [57]. In cancer research, it was found that the upregulation of NFIX causes poor prognosis due to its association with ROS [58]. The Nfix expression is significantly upregulated in the current study, which demonstrates the regulation of ROS is disrupted. ELP6 is one of the autophagosome regulatory genes, also maintaining the homeostasis of the number of retinal cells. which is found to be upregulated under inflammatory stress conditions. Nascent polypeptide-associated complex subunit alpha (NACA) prevents ER stress by binding to the nascent polypeptides and blocking their interaction with the signal recognition particles [59]. In the current study, Naca showed higher expression upon ER stress induction. And Plcd3 is downregulated under hypoxic conditions.

Thus, the RNAseq data revealed the effects of each type of stress stimulus at a whole retina scale and illustrated how the retinal cells respond in concert. While the initial insult in any retinal disease may originate from a specific subset of cell types, however, the visual response is usually more involved across layers upon disease progression to the retinal degeneration stages. Often, when patients present at the clinic, photoreceptors as well as RPE and support cells are already degenerated in specific areas with other areas in transition depending on the nature of the disease. We find that in most stresses, pyroptosis emerges as the most dysregulated mechanism in ER stress, oxidative stress and inflammatory stress with pyroptosis and extrinsic apoptosis being critical in hypoxia. Thus, pyroptosis is a favored mechanism in the whole retina which is now supported by the additional meta-analysis performed in available RNAseq datasets from other retina models. Therefore, it is likely that in most human retinal diseases, the degenerating retina, with multiple stresses operational, pyroptosis or ferroptosis are important mediators of cell death. This is an important observation from the point of view of the field which can now potentially investigate such common pathways for prevention of retinal degeneration as a therapeutic modality. Importantly, future studies on the signaling mechanisms underlying the new genes and favored cell death mechanisms may lead to identification of specific inhibitors that can prevent retinal degeneration in a broad range of diseases. Such applications may have critical impact on rare diseases like RP where, although the genetic basis is diverse, the phenotype shows overlap, particularly in the degeneration of the retinal layers.

However, there are a few limitations of the study. The acute treatment model adopted in the current study administered a fixed amount of dosage at a single time point. The chronic, prolonged exposure model could be useful in studying the progressive loss of retinal cells, follow-up studies that we have planned. The cell death mechanisms were derived from specific gene sets curated from the literature and relevant databases, which may be improved in the future as more functional data accumulates from experimental models. Taken together, the data deepens our understanding of stressor-induced cell death mechanisms in the retina.

## Supplementary information

Supplementary table 1a

Supplementary table 1b

Supplementary table 1c

Supplementary table 1d

Supplementary figure 1

Supplementary figure 2

## Data Availability

The transcriptomics data is available at NCBI-SRA under project ID PRJNA1255768.
