# Supplementary figures and images for: Comparative transcriptomic analysis of retinal response to diverse cellular stresses reveals relative contributions of different cell death processes and signalling networks
Source: Cell Death Dis. 2025 Dec 1;16(1):876. doi: 10.1038/s41419-025-08257-w (PMC12669722; doi:10.1038/s41419-025-08257-w)

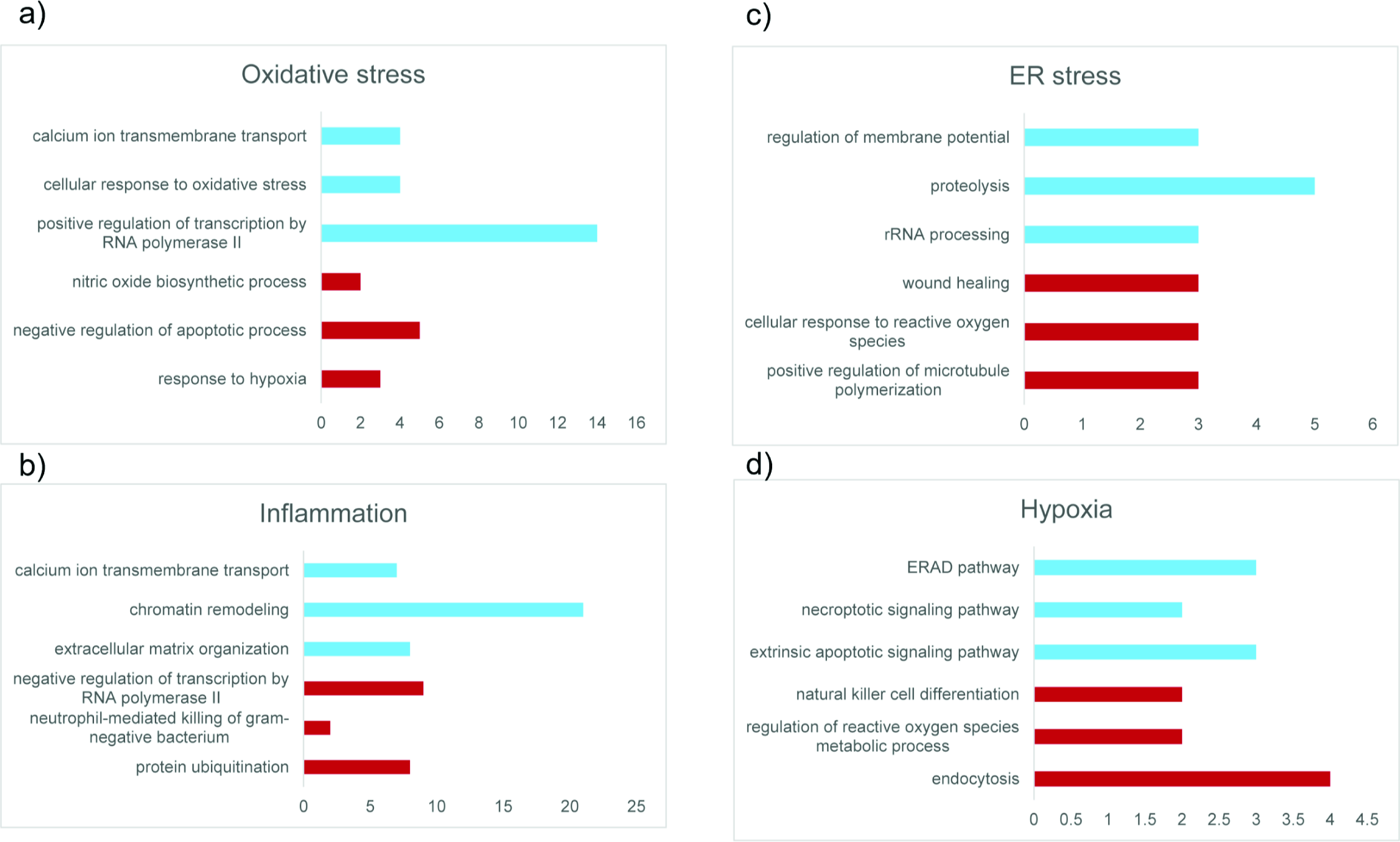

Supplement: Supplementary file 5 — Supplementary figure 1 [file 41419_2025_8257_MOESM5_ESM.tif]

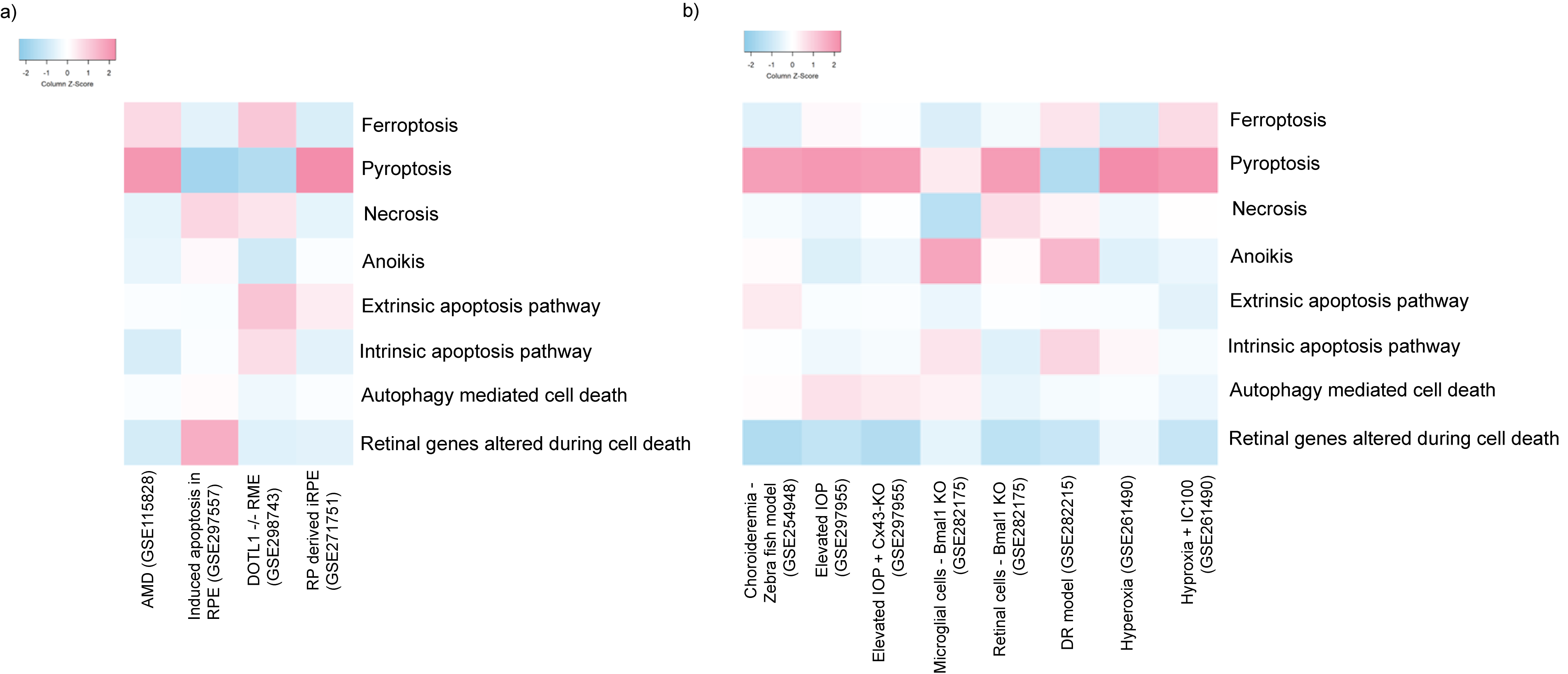

Supplement: Supplementary file 6 — Supplementary figure 2 [file 41419_2025_8257_MOESM6_ESM.tif]
